# Remarks on Syphilization

**Published:** 1858-02

**Authors:** 


### Remarks on Syphilization.

We find in the *Gazette Hebdomidoire* the following extract of a letter from M. Guepin, of Nantes, to the *Jour. des Conu. Med.*, 2d Oct., 1857 :

"Syphilization is practiced in some parts of India upon a grand scale, as a cause as well as a means of preventing Syphilis. Often the prostitutes of India proposed to practice syphilization upon our sailors, which they invariably refused, objecting to any method that was not adopted in Europe. The officer who gave this statement was excited by curiosity to see some of the women who made this proposition to the sailors; he discovered no disease of the skin upon any one of them, nor was he able to detect any cicatrix that could have resulted from a bubo. This fact, he adds, is of more importance as they were not apprised that syphilization had been practiced with success in France, at Turin, and at Christiana. Again, he remarks that syphilis contracted in India is of the very worst form. The French who contract the disease there frequently fall victims to its ravages, or if they are cured requiring the most energetic and methodic treatment for a length of time.

